# Active dispersal of *Aedes albopictus*: a mark-release-recapture study using self-marking units

**DOI:** 10.1186/s13071-019-3837-5

**Published:** 2019-12-12

**Authors:** Laura Vavassori, Adam Saddler, Pie Müller

**Affiliations:** 1 Swiss Tropical and Public Health Institute, Socinstrasse 57, P.O. Box, 4002 Basel, Switzerland; 2 University of Basel, Petersplatz 1, P.O. Box, 4001 Basel, Switzerland; 3 Ifakara Health Institute, Environmental Health and Ecological Sciences, P.O. Box 74, Bagamoyo, Tanzania

**Keywords:** Population dynamics, Invasive mosquitoes, Flight range, Mosquito survival

## Abstract

**Background:**

Understanding the dispersal dynamics of invasive mosquito species is fundamental to improve vector surveillance and to target control efforts. *Aedes albopictus* has been deemed a poor flyer as its range of active dispersal is generally assumed to be limited to a few hundred metres, while laboratory studies suggest this mosquito could actually fly several kilometres. The discrepancy may be due to differences in the local environment or to the methodological approach. In Switzerland, *Ae. albopictus* has been present since 2003 and has since then expanded its range. While passive dispersal is a key driver, it remains unclear how far this mosquito spreads through active flight and what the age structure and size of the local population are, all important parameters for vector surveillance and control.

**Method:**

We investigated the active dispersal, daily survival rate and population size of *Ae. albopictus* in mark-release-recapture studies in Coldrerio and Lugano, two areas of intensive control in Switzerland. To mark mosquitoes emerging from breeding sites, we used self-marking units with fluorescent pigment that have minimal impact on mosquito survival and behaviour. We recaptured the adult mosquitoes with BG-Sentinel traps within a radius of 1 km from the marking units over 22 consecutive days.

**Results:**

We found that 77.5% of the recaptured *Ae. albopictus* individuals flew further than 250 m, the limit that is usually deemed sufficient for vector containment. The average age of females and males was 8.6 days and 7.8 days in Coldrerio and Lugano, respectively, while the estimated mosquito population densities were 134 mosquitoes/ha in Coldrerio and 767 mosquitoes/ha in Lugano.

**Conclusions:**

Self-marking units are an effective tool to mark wild mosquitoes. Using this approach, we found that mosquitoes survive long enough to potentially transmit arboviral disease in our study area and that host-seeking *Ae. albopictus* females may travel further than previously assumed for European mosquito populations. This finding has direct implications for vector control as emergency treatments around positive cases, as well as surveillance and control around detections of new infestations, might need to be extended beyond the usual recommended range of just a few hundred metres
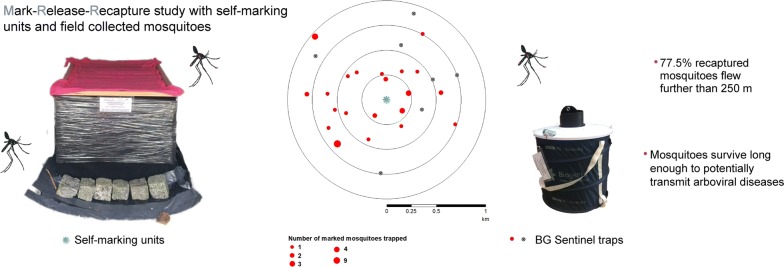
.

## Background

The Asian tiger mosquito, *Aedes albopictus* (Skuse, 1894) is listed as one of the most invasive mosquito species worldwide [1]. Besides the considerable biting nuisance, *Ae. albopictus* is a vector of several pathogens, including chikungunya, dengue and Zika virus, as well as dirofilarial worms [2, 3]. With its global spread and an increasing number of infected travellers returning from disease-endemic countries, outbreaks of tropical and subtropical mosquito-borne diseases have become a reality also in Europe, exemplified by the autochthonous cases of chikungunya in Italy [4, 5] and France, and dengue in Croatia, France and Spain [6–11] with *Ae. albopictus* identified as the incriminated vector.

Due to desiccation resistant eggs, *Ae. albopictus* is passively spread across the globe through the international trade of used tyres and other artificial containers. At a more regional scale, adults are travelling as blind passengers in vehicles and are dispersed particularly along the main traffic routes [12–14]. In contrast, *Ae. albopictus* has been deemed a poor flyer as its range of active dispersal is generally assumed to be limited to a few hundred metres (Table 1), while laboratory studies suggest the mosquito could actually fly several kilometres [15], leaving some uncertainties as to whether its actual flight range might have been underestimated.

**Table 1:** Summary information of MRR studies conducted with *Ae. albopictus*

Study	Continent, country, location	Mosquito source	Marking	No. released	Recapture method	Trapping effort	Recapture rate (%)	MAX (m)	MDT (m)	FR_90_ (m)
[44]	Europe, Italy, Castel Maggiore	Laboratory colony	FD	1700 ♂♂	Aspiration	4–5 people, 9 h over 3 days	1.5	218	148	119
			WB0	920 ♂♂			2.9	196	97	162
	Europe, Italy, Altedo		FD	3600 ♂♂			0.6	236	115	175
			WB0	2100 ♂♂			2.4	323	203	309
	Europe, Italy, Castello d’Argile		FD	2000 ♂♂			4.7	238	109	176
			WB0	1600 ♂♂			11.1	312	212	307
Present study	Europe, Switzerland, Coldrerio	Field population	FD	427 ♂♂ + ♀♀	BG-sentinel (no CO_2_)	8.9 traps/km^2^, over 22 days	9.3	960	631	826
	Europe, Switzerland, Lugano		FD	425 ♂♂ + ♀♀		8.3 traps/km^2^, over 22 days	2.1	977	685	861
[45]	Europe, Italy, Rome	Reared from field-collected eggs	FD	1582 blood-fed ♀♀	Sticky trap	^a^278 traps/km^2^, over 21 days	4.5	199	105	168
								168	118	191
								290	154	236
[46]	Africa, La Réunion, Saint Pierre	Laboratory colony from field-collected eggs	FD	704 ♂♂	BG-sentinel (no CO_2_-mice)	^a^2500 traps/km^2^	13.4	≥ 50	na	na
				813 ♂♂ + ♀♀		^a^889 traps/km^2^, 3 weeks	16.5	100	na	na
[50]	Africa, La Réunion, Sainte Marie	Field-collected eggs	FD	2493 ♂♂	BG-sentinel (no CO_2_-mice)	20 traps in 0.0707 km^2^ over 4 days of collection; 3Rep	5.9	≥ 150	46	na
				2731 ♂♂			5.6		67	na
				1453 ♂♂			11.6		37	na
		Laboratory colony	FD	2589 ♂♂			4.1		65	na
				1497 ♂♂			11.9		42	na
[49]	Africa, La Réunion, Saint Pierre	Laboratory colony from field-collected eggs	FD	913 ♀♀	BG-sentinel (no CO_2_-mice)	^a^637 traps/km^2^, 4 × 6 days	9.4	≥ 100	na	na
				1914 ♂♂			7.2	≥ 100	na	na
[36]	North America, Texas, Central Texas	Field population	^13^N, ^13^C	1003 ♂♂ + ♀♀	CDC gravid + CDC light + BG-sentinel	80 traps per 78 locations over 5 months	18 ♀♀	737	300	na
							2.3 ♂♂	33	300	na
							3.8 ♀♀	656	400	na
							3.5 ♂♂	1900	1100	na
[60]	North America, Hawaii, Oahu	Laboratory colony	CD	7100 ♂♂ + ♀♀	HLC	na	3.8	430	na	na
[28]	North America, Missouri	Field-reared adults	FD	13,513 ♂♂ + ♀♀	Aspiration	16 days	8.1	525♀♀	na	na
								225♀♀		
[54]	North America, FL, Gainsville	Field-caught adults	FD	na	Sticky ovitrap	50 traps/250 m over 32 days	9.3	149	58–78	na
[48]	South America, Brazil, Rio de Janeiro	Laboratory colony from field-collected immatures	FD	2689 ♂♂ + ♀♀	Aspiration + Ovitrap	140 min every 3rd day	1.6	≥ 1250	na	na
			RB	774 ♀♀	Ovitrap	70 traps along 1 km trail over 34 days	na	≥ 1000		
[61]	South America, Brazil, Nova Iguaçu	Laboratory colony	RB	2225 ♀♀	Ovitrap	^a^183 traps/km^2^, 6 days	na	800	na	na
[51]	Asia, Japan, Nagasaki	Laboratory colony; field population	IM	706 ♀♀	HLC	10 collection points during 13 days	13.0	na	na	na
				150 ♀♀			20.7			
[52]	Asia, Japan, Ishigaki	Field-collected adults	IM	232 ♀♀	HLC	4 collection points over 9 days	20.7	≥ 187	na	na
[22]	Asia, Japan, Nagasaki	Laboratory colony (ld)	FD	1000 ♀♀	HLC/ Aspiration	16 days	15.4	≥ 260	na	na
				1000 ♂♂		14 days	10.6			
				1500 ♀♀			5.9			
				1000 ♂♂			7.2			
[62]	Asia, Singapore, Bidadari Geylang	Laboratory colony	RB	100 gravid ♀♀	Ovitrap	^a^33 traps/km^2^	na	≥ 320	na	na
				100 gravid ♀♀		^a^23 traps/km^2^, 4 days				
[63]	Oceania, French Polynesia, Taiaro	3 laboratory colonies	None	6000–12,000 ♂♂ + ♀♀ of each colony	HLC, nets and larval sampling	3 weeks	na	≥ 600	na	na

^a^Recalculated based on published data

*Abbreviations*: FD, adult mosquitoes marked by fluorescent pigment; CD, adult mosquitoes marked by coloured pigment; hd, larvae reared at high density (4000/tray); HLC, human landing catches; IM, individual marking of wings or thorax; ld, larvae reared at low density (100/tray); RB, females fed with rubidium which accumulates in the eggs; WB0, adult males marked by removing *Wolbachia*; ^15^N, ^15^N stable isotope enrichment; ^13^C, ^13^C stable isotope enrichment; MAX, maximum dispersal distance; MDT, mean distance travelled; FR, flight range

Understanding the dispersal dynamics of invasive mosquito species is fundamental to improve vector surveillance and to target control efforts. Whether being used to control a new or existing mosquito infestation, the dispersal potential of a mosquito species will have important implications for the spatial scale at which control interventions need to be deployed. For example, if a returning traveller presents viremia in an area where the local mosquito population is competent to transmit the virus, control measures may be deployed within the vector’s flight range in order to minimise the risk of further spread. Current guidelines recommend vector control measures to be implemented within a radius of 100 m from the residence of a suspected or confirmed case or 300 m of a cluster of cases [16–19].

Active dispersal, population size and other mosquito population related parameters have frequently been investigated in mark-release-recapture (MRR) studies, where adult mosquitoes are coloured with fluorescent pigment, released and then recaptured in traps [20, 21]. In most studies with *Ae. albopictus*, the mosquitoes were sourced from laboratory colonies that had been reared over many generations (Table 1). However, rearing conditions have a strong influence on mosquito physiology, which will also influence dispersal behaviour [22]. Additionally, mosquitoes have mostly been individually marked with tedious and time-consuming methods, involving direct manipulation of mosquitoes, casting doubt on the validity of such MRR studies.

In Switzerland *Ae. albopictus* was first detected in 2003 at a motorway service area [23] and since then has continued to spread across larger areas of the Canton of Ticino [13]. Despite an intensive control programme based on public awareness campaigns, larval source reduction and larviciding, the mosquito has spread across many areas in Ticino. Nevertheless, relative population densities have been shown to be twice as high in non-intervention areas in neighbouring regions in Italy, supporting the hypothesis that the vector control efforts still have a major impact on mosquito density [24].

In this study we aimed at estimating the dispersal patterns of *Ae. albopictus* in an area where control measures are in place, while overcoming the drawbacks of commonly used methods to mark released mosquitoes. Our results provide the first data on active dispersal in Switzerland and we discuss them in the framework of risk assessment of disease transmission and effective vector control.

## Methods

### Study sites

**Fig. 1:**
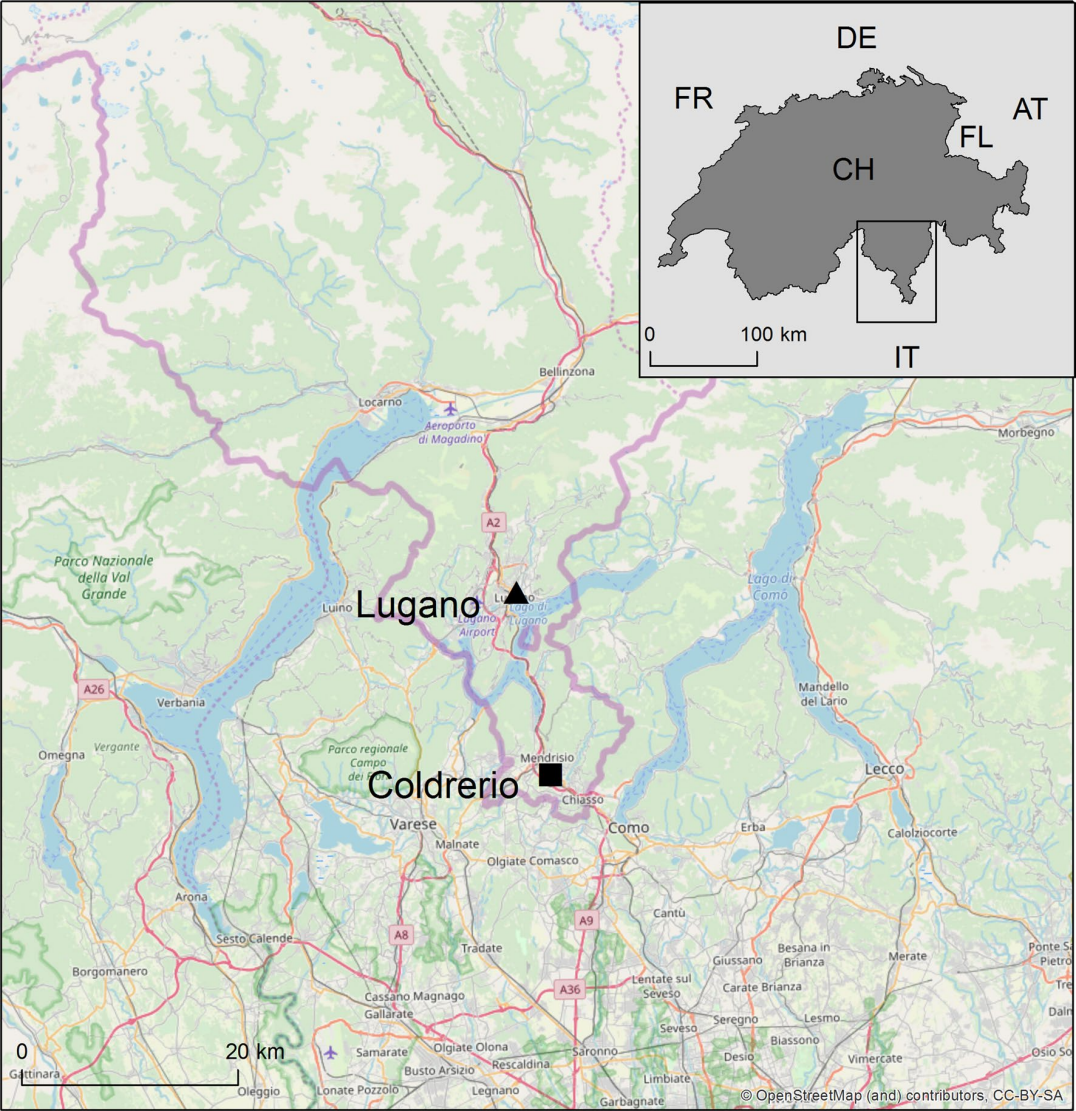
Study sites of the two MRR experiments in southern Switzerland. *Abbreviations*: AT, Austria; CH, Switzerland; DE, Germany; FR, France; FL, Lichtenstein; IT, Italy. Map source: OpenStreetMap and contributors CC-BY-SA

We conducted our MRR experiments at two sites in southern Switzerland in the Canton of Ticino, Coldrerio and Lugano (Fig. 1) from 9th to 31st of August and from 8th to 28th of September 2018, respectively. These dates coincide with the peak of the *Ae. albopictus* season in Ticino [25].

Coldrerio (45°51″N, 8°59″E, 351 m) is a municipality in the district of Mendrisio and in 2017 had a population of 2892 inhabitants [26]. Coldrerio covers an area of 246 ha of which 20% (47 ha) are woods, 50% (124 ha) are agricultural areas and 30% (74 ha) are urban. The landscape is representative for a municipality of southern Switzerland, where the land is primarily used for agricultural purposes (i.e. mainly vineyards). The highway E35, connecting Rome with Amsterdam, crosses the municipality of Coldrerio where the first specimens of *Ae. albopictus* in Switzerland were found in an ovitrap placed at the motorway service station in 2003 [23].

Lugano (46°00″N, 8°57″E, 273 m) is the largest city in the canton and had a population of 63,932 in 2017 [26]. Lugano city centre lies on Lake Lugano and is surrounded by two mountains, Monte Brè (925 m) and San Salvatore (912 m) that overlook the lake. Lugano covers a surface of 30,811 ha, of which 66% (20,288 ha) are covered by forests, 12% (3654 ha) are agricultural areas, 17% (5126 ha) are urban land and 5% (1730 ha) are covered by water.

### Self-marking units

**Fig. 2:**
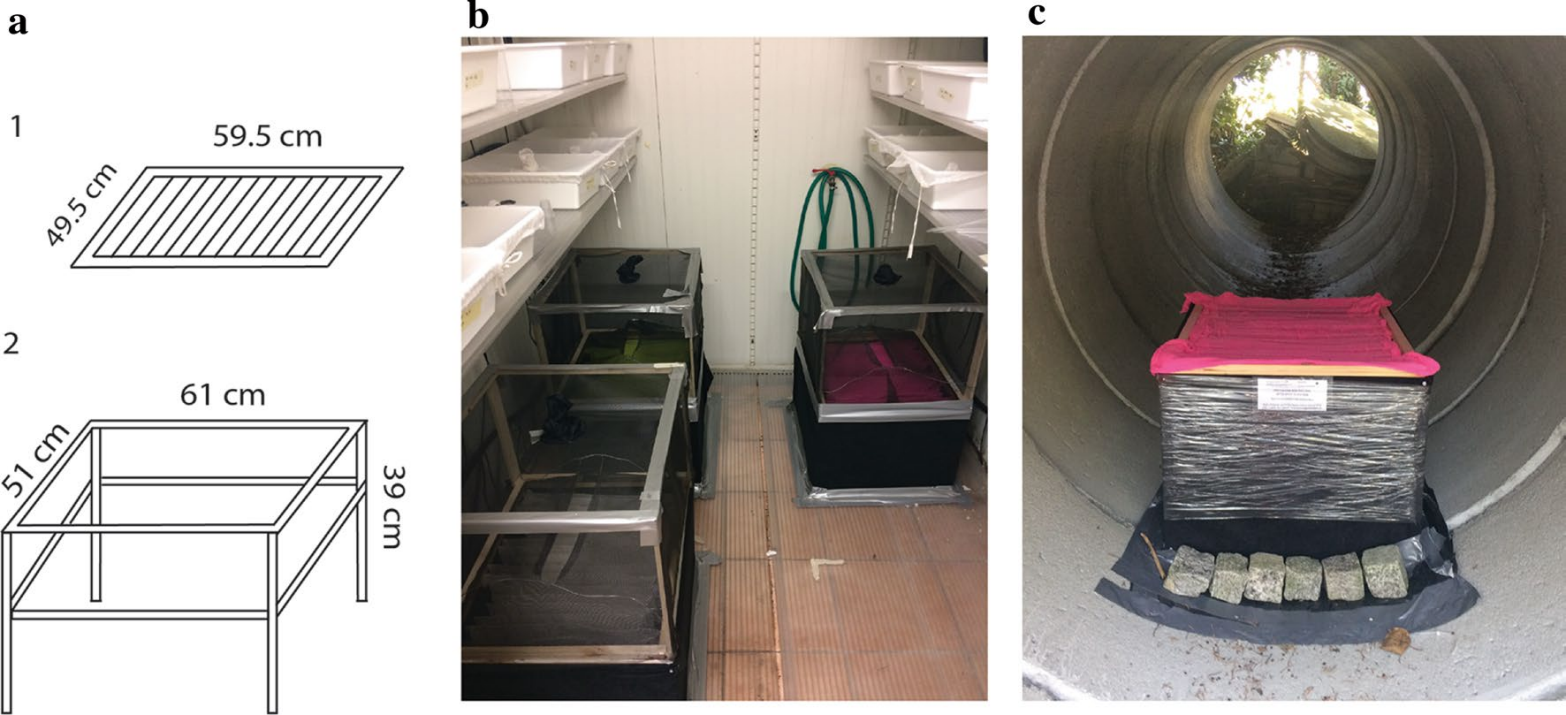
Mosquito self-marking unit. **a** The self-marking unit comprises a removable exit grid (1), supporting cheese cloth stripes impregnated with fluorescent pigment, which is mounted on top of a metal structure (2). **b** Self-marking units with exit traps mounted on top of the grid with the cheese cloth. A funnel constructed of wire and mesh restricts the mosquitoes to fly only in one direction. **c** Self-marking unit placed in the field. The plastic foil prevents lizards and other animals from entering the unit

We investigated the active dispersal behaviour of *Ae. albopictus* using a unit in which mosquitoes emerging from a breeding site mark themselves with fluorescent pigment (Fig. 2). The unit was originally developed for *Culex* mosquitoes [27] and has also already been deployed in a study to mark emerging *Ae. albopictus* [28] in the field.

In the present study, the self-marking units consisted of three parts: an aluminium structure, a removable exit grid and cheese cloth strips of 55 × 20 cm that are impregnated with fluorescent pigment (Fig. 2). The aluminium structure supports the removable exit grid that is made of a wooden frame and aluminium rods while the colour-pigmented cheese cloth strips are suspended and kept in place by the rods. Upon emergence from trays placed underneath the unit, the freshly emerged adult mosquitoes have to find their way through the pigmented cloth before they can fly off, picking up fluorescent pigment particles. We attached black fabric to the sides of the aluminium structure with Velcro, closing the unit so that mosquitoes could only exit through the impregnated cloths. In each study site, we deployed two self-marking units and placed them next to each other at the centre of the study area.

### Evaluation of the self-marking units under laboratory conditions

To evaluate the impact on the marking success and potential impact on mosquito survival [29], we conducted preliminary experiments to evaluate the effectiveness of two different dyes of fluorescent pigment, “TP-48: Magenta” and “TP-40: Chartreuse” (RadGlo^®^ TP, Radiant Color N.V., Houthalen, Belgium), and their impact on the survival of field-caught *Ae. albopictus.* We performed the experiments under controlled laboratory conditions with 28 ± 1 °C, 80% relative humidity and a 14:10 h day:night photoperiod. We then compared the performance and impact on mosquito survival of the two fluorescent pigments alongside a negative control (i.e. cheese cloths without pigment). The two colours were chosen, because these have been shown to be the least (TP-48: Magenta) and the most (TP-40: Chartreuse) visible ones for marking *Anopheles arabiensis* (Adam Saddler, personal observation).

In the experiment, we placed 60 laboratory-reared *Ae. albopictus* pupae, that emerged from eggs collected in Parma in 2016 (see [30] for rearing description), in a tray to hatch under each unit. Mosquitoes were left under the units for three consecutive days to give them time to emerge and fly upwards into exit traps we had mounted on top of the marking units to catch the marked mosquitoes (Fig. 2b). From the exit traps we then transferred the adult mosquitoes individually with a mouth aspirator into collection cups covered with a black net (TESA SA manufactures, Renens, Switzerland). Mosquitoes were provided with water only, and on each day we recorded the number of dead mosquitoes. We inspected the dead mosquitoes under a stereo microscope with a UV light (60 LED Purple UV light source for Microscope, Nanyang Srate Optical Instrument Co., Ltd, Nanyang City, China) for the presence or absence of fluorescent pigment. We repeated the experiment three times with different batches of mosquitoes while rotating the positions of the units.

### Larval collections

Across each study area we sampled the mosquitoes as late third- and fourth-instars because these stages allow for morphological identification of *Ae. albopictus* [31]. The larvae were collected within 3 km from the marking units, together with the water from their breeding sites, and we transferred them to circular, 13 cm wide plastic containers containing approx. 350 ml of water and left them to pupate. Initially, the intended area of larval collection was within the 1 km radius of the study areas, but in order to collect enough larvae we enlarged the search radius to 3 km away from the marking units. Larvae collected from breeding sites at different time points were kept in separate containers and then placed under the self-marking units once at the pupal stage. For quality control of species identification at the larval stage we put aside a subset of mosquitoes, hatched them out and identified them at the adult stage. We calculated the number of marked mosquitoes by subtracting the number of pupae and adults remaining in the unit from the number of pupae initially placed under the unit on day 0.

### Colour-marking of adults

We dissolved the fluorescent colour pigment in tap water, soaked strips of cheese cloth in the solution and then let it dry overnight. To avoid cross-contamination we coloured the cheese cloths in a separate laboratory from the one where we screened the mosquitoes for fluorescent pigment particles. In order to investigate the age structure of the local *Ae. albopictus* population, we used four different colours and swapped between them every 4th day. The colours were chartreuse (TP-40), followed by magenta (TP-48), red (TP-45) and orange (TP-43). That way the marked mosquitoes could be traced back to the 4-day period in which they emerged from the marking units. We had chosen a time period of four days based on preliminary experiments, leaving enough time for the mosquitoes to emerge from the pupae and fly upwards through the marking unit.

### Mosquito recapture

**Fig. 3:**
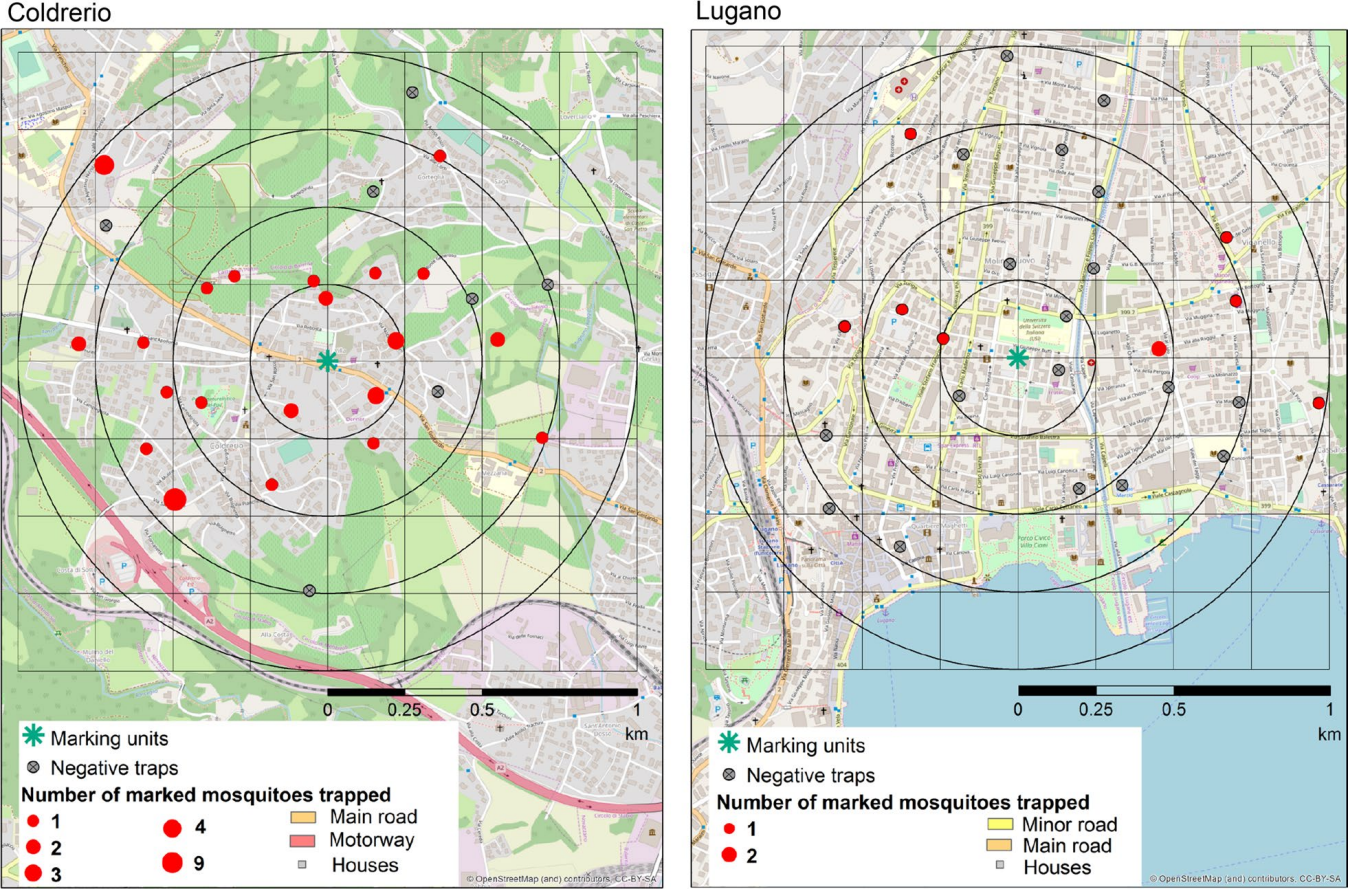
Map of the MRR set-ups in Coldrerio and Lugano. The traps were deployed in all directions from the self-marking units (green star in the centre) across a circular area with a radius of 1.0 km. Red dots represent positive traps while the size of the dots corresponds to the number of marked mosquitoes caught at each site. Grey dots represent negative traps. Circular black lines represent the four annuli at 250, 500, 750 and 1000 m from the marking units. Basemap source: OpenStreetMap and contibutors CC-BY-SA

We recaptured the adult mosquitoes with BG-Sentinel 2 traps (Biogents, Regensburg, Germany) within a radius of 1 km from the marking units. In order to analyse the distance travelled by the mosquitoes from the marking units, we geo-referenced each trap with a hand-held GPS device (Garmin eTrex 20X, Garmin Ltd, Southampton, UK). An attempt was made to have an even distribution of 16 traps per km^2^ (i.e. 1 trap within a 250 m by 250 m square); however, this was not feasible to implement due to several constraints in the field. Eventually we managed to deploy 28 BG-Sentinels in Coldrerio and 26 in Lugano yielding an overall trap density of 8.9 traps/km^2^ and 8.3 traps/km^2^, respectively (Fig. 3). All traps, except one in each site, were connected to the main power supply, while the reaming two traps were powered by a battery (NP12-12 Lead-Acid Battery 12 V 12 Ah, Yuasa, Kyoto, Japan). In Coldrerio, the trap closest to the release point was placed 194 m and the furthest 960 m from the self-marking units. In Lugano, the distances were 138 m and 977 m, respectively. We equipped the BG-Sentinels with BG-Lure cartridges (Biogents) but operated them without CO_2_. As *Ae. albopictus* males seek females near the host, they may also be caught with BG-Sentinels [32].

A cause of concern was cross-contamination from marked to unmarked mosquitoes within the same trap, which would lead to unwanted biases in the number of marked mosquitoes. To avoid such cross-contamination, we slightly modified the BG-sentinel traps. The collection bags were wrapped around a cylinder-shaped chicken wire that supported sticky cards (Additional file 1: Figure S1) originally developed for the use in BG-GAT traps (Biogents). The sticky cards immobilised any mosquito inside the bag, preventing the transfer of colour particles from one individual to another. We had previously validated this modification in a laboratory experiment with *Ae. aegypti* mosquitoes that were released into a 14 m^3^ free-flight chamber into which we placed such a modified BG-Sentinel trap.

In the field experiments, we replaced the sticky cards from the BG-Sentinels every other day over a period of 22 days from the day the pupae were placed under the marking units and transported the cards inside transparent plastic folders to the laboratory for morphological identification.

### Weather parameters

Weather parameters, including temperature, relative humidity, wind speed and direction, and precipitation were recorded throughout the study. For Coldrerio we obtained the weather data from AgroMeteo [33] from a station located 600 m from the marking units. In Lugano we deployed a professional weather station (Vantage Pro 2, Davis instruments, Hayward, CA, USA) 2.4 km away from the marking units near the laboratory. In addition, each BG-Sentinel was equipped with an USB data logger (HOBO^®^ Pendant^®^ Temperature/Light Data Logger, Onset Computer Corporation, Pocasset, MA, USA) that recorded temperature and light intensity every hour at the top of the trap.

### Data analysis

We analysed the effect of the marking on the survival of mosquitoes using the Kaplan–Meier method that compares survival curves from the unmarked (control) and the marked cohorts by the log-rank test. The dispersal patterns are described by the mean distance travelled (MDT), the maximum distance travelled (MAX) and the flight range (FR) for each locality according to Morris et al. [34]; their formula for MDT includes a correction factor accounting for differences in trap density. For MDT calculation, we divided the circular area into 4 concentric annuli, with a width of 250 m. We calculated the FR on the basis of a linear regression model of the cumulative estimated recapture in the 4 annuli as described in [35]. FR_90_ represents the maximum flight distance reached by 90% of all individuals. To estimate the accuracy of the MDT and FR estimation, we calculated a range for the estimates by removing one trap at a time for each location and repeating the calculation for every possible combination. The detection probability was calculated accounting for trapping effort and the area of the annulus [36]. We estimated survival rates with a general nonlinear regression approach as outlined by Buonaccorsi et al. [37] which adjusts for mosquito removal as a result of recapture. Confidence intervals of the survival estimate were calculated by bootstrapping (1000 repeats) using the *nlstools* package in R [38, 39]. For the average life expectancy, we used the formula from Niebylski & Craig [28]. Population size estimates were calculated using the Lincoln index modified by Bailey. We produced the maps with ArcMap 10.5 (ArcGis 10.0, ESRI Inc., USA) and the graphs with the R package *ggplot2* [40]. For the statistical tests we set the level of significance at *α* = 0.05.

## Results

### Preliminary experiments

The preliminary laboratory study revealed that the self-marking units mark between 65% (magenta) and 89% (chartreuse yellow) of the emerging *Ae. albopictus* and that the survival of the marked mosquitoes is statistically not significantly different to the unmarked mosquitoes (*χ*^2^ = 2, *df* = 2, *P* = 0.133), neither for female nor male mosquitoes (female: *χ*^2^ = 4, *df* = 2, *P* = 0.1; male: *χ*^2^ = 1.6, *df* = 2, *P* = 0.4, Additional file 1: Figure S2).

As the marking success did not reach 100%, we changed the size of the cheese cloth strips to one single piece with a total length of 6.3 m^2^ folded between the rods in order to increase the coloured surface area and, therefore, the marking success. We tested this modification with *Ae. aegypti* mosquitoes and achieved a minimum marking effectiveness of 82% (*n* = 75).

### Mosquito release and recapture

We released 521 colour-marked *Ae. albopictus* in Coldrerio and 519 in Lugano. Taking the marking effectiveness from the preliminary experiments into account, we estimated that 427 mosquitoes have been marked in Coldrerio and 425 in Lugano. While in Coldrerio 40 mosquitoes were recaptured, only 9 mosquitoes were recaptured in Lugano giving an overall recapture rate of 9.3% and 2.1%, respectively. In Coldrerio we collected a total of 3970 *Ae. albopictus*, 2537 females (64%) and 1433 males (36%), and in Lugano a total of 4894, 1994 males (40.7%) and 2900 females (59.3%). The detailed results from the mosquito recaptures are reported in Additional file 1: Figure S3 and Table S1.

The distribution of the marked mosquitoes in the two MRR experiments is represented in Fig. 3. In Coldrerio, marked mosquitoes were recaptured in all direction from the marking units (Fig. 3a); 25% of the traps (*n* = 28) did not catch marked mosquitoes.

### Dispersal

**Fig. 4:**
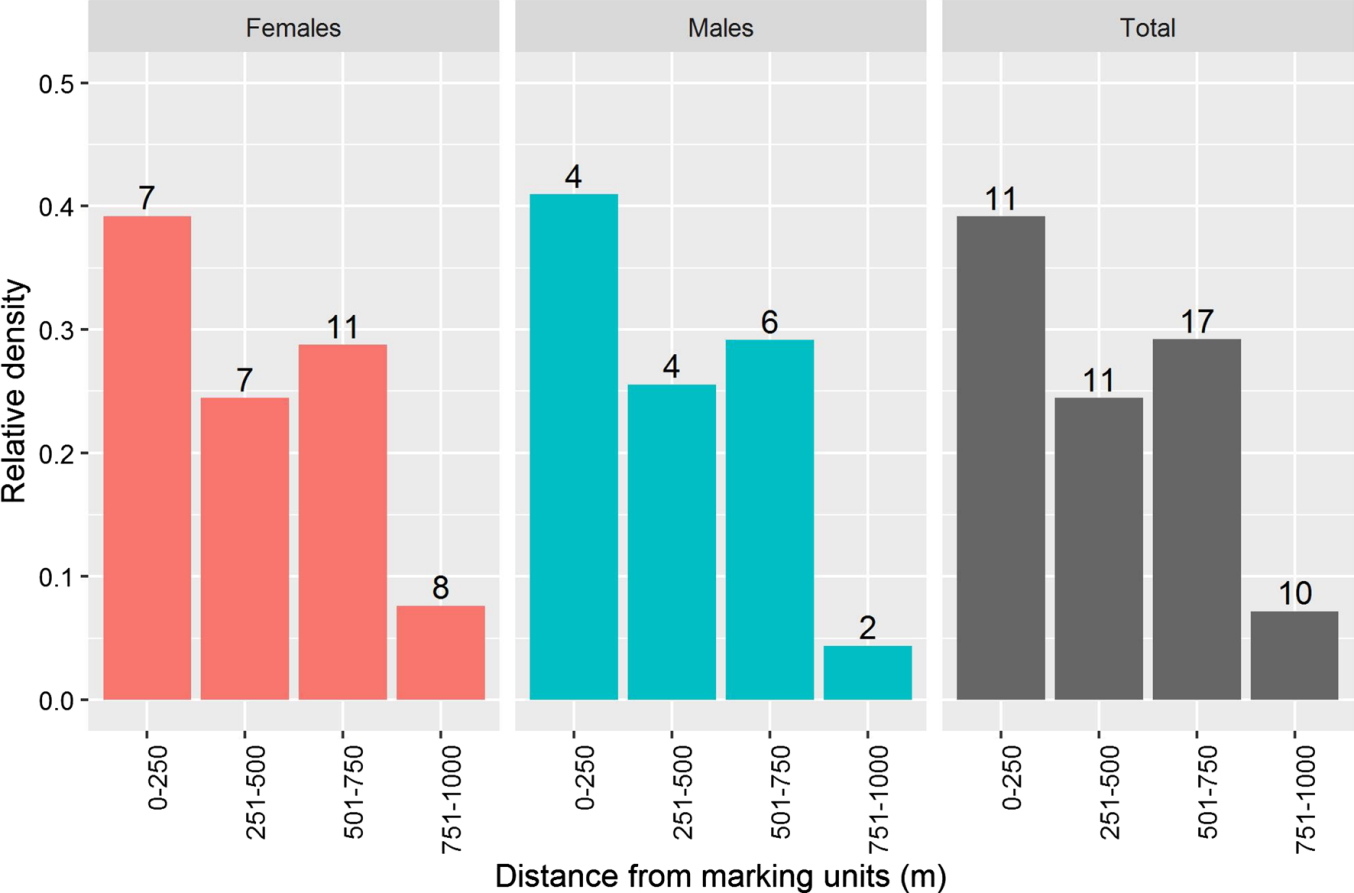
Estimated relative mosquito density corrected for the area of the annulus and trapping effort. Data from the two MRR sites were pooled. The numbers of mosquitoes caught are indicated on top of the respective bars

The calculated FR_90_ is 826 (778–849 m) and 861 m (761–866 m) in Coldrerio and Lugano, respectively (average: 843.5 m). The calculated MDT is 631 m in Coldrerio ranging from 576 to 648 m, and 685 m in Lugano, ranging from 661 to 737 m, respectively. The additional dispersal parameter, such as MAX, is reported in Table 1. To calculate the corrected relative mosquito density, we pooled data from the two sites and found that 93% of the mosquitoes (*n* = 49) were recaptured within 750 m from the marking units (Fig. 4). No difference was observed between females and males (Fig. 4). The observed pattern does not reflect a typical decay curve in density away from the marking units within our 1 km radius, and a total of 38 marked mosquitoes (77.5%) were recaptured more than 250 m away from the self-marking units.

**Fig. 5:**
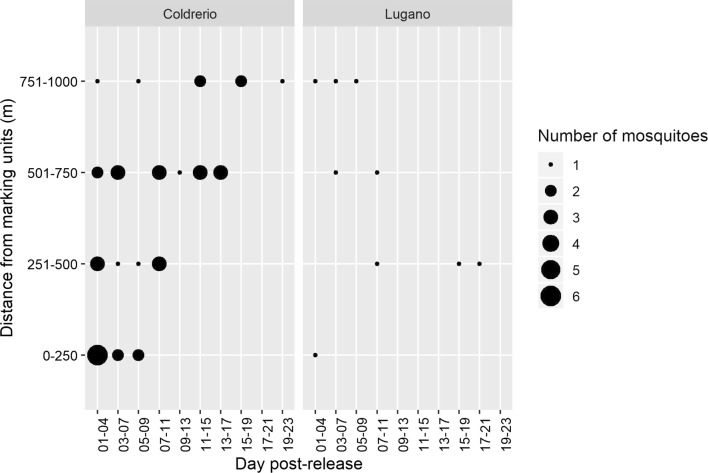
Number of mosquitoes recaptured as a function of the time following marking. The size of the dots corresponds to the number of marked mosquitoes caught. The number of marked mosquitoes can be traced back to 4-day periods as the different pigments were swapped every fourth day. Additionally, the recapture was conducted only every other day. The day post-treatments consists in a window of four days (i.e. 01–04 means from day 1 to day 4)

Based on the number of mosquitoes recaptured as a function of time following their release, the estimated dispersal rate is between 83 and 333 m/day (Fig. 5, Additional file 1: Table S1). While we did not find a clear pattern in the results from Lugano, for Coldrerio we observed a distinct relationship between the number of marked mosquitoes captured and the day after their release (Fig. 5). The majority of the mosquitoes were recaptured within the first 7 days after marking and did not fly beyond the 750 m mark. In contrast, the mosquitoes recaptured between 8 and 11 days were caught in the marking units placed within the two outer annuli.

### Survival

**Fig. 6:**
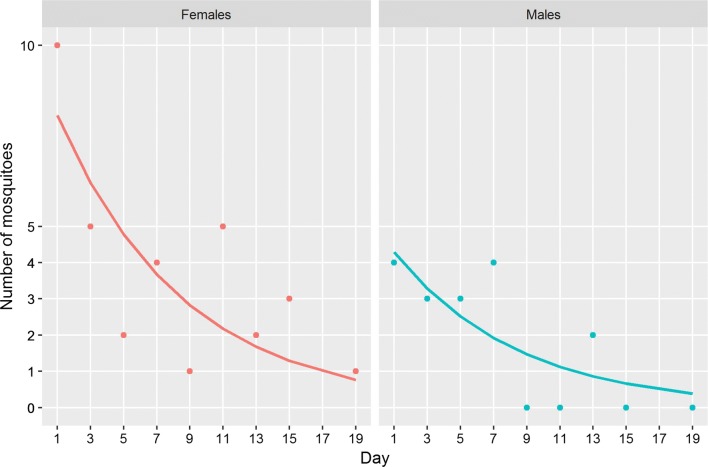
Daily survival of female and male *Ae. albopictus*. The points show the total number of mosquitoes caught in the BG-Sentinel traps as a function of age. The line shows the estimated average survival. Data from both study sites were pooled

To calculate survival rates, we pooled the data from both study sites. On that basis we estimated that females live on average 8.6 and males 7.8 days (Fig. 6). For the females the average daily survival rate was 0.89 (95% CI: 0.80–0.94), while the daily survival rate for males was 0.88, (95% CI: 0.77–0.97) suggesting that 25% of female *Ae. albopictus* reach an age of 12 days, an age of concern for transmission scenarios considering the incubation period of dengue and chikungunya [41], considering the fact that after emergence mosquitoes need a few days until mating and their first blood meal.

### Population size

The population density in Coldrerio was five times lower than in Lugano as estimated with the modified Lincoln index [42]. In Coldrerio the estimated density was 41,953 mosquitoes within the MRR area during the study period, which corresponds to 134 mosquitoes/ha. In contrast, for Lugano the number of mosquitoes was estimated at 208,038 mosquitoes, corresponding to 767 mosquitoes/ha. The estimates are corrected for the area of the lake overlapping with the 1 km circle in Lugano. In Coldrerio, we calculated additionally the build area and observed that the estimate would double from 134 mosquitoes/ha to 282 mosquitoes/ha, assuming they are predominantly present in the built environment.

### Weather parameters

In Coldrerio, the data loggers on the BG-Sentinels recorded temperatures ranging between 10.5–56 °C, with a mean of 24.9 °C. As already observed by Crepeau et al. [43], exposure of data loggers to sunlight caused different spikes in the temperature recorded, leading to abnormalities in the maximum temperature registered. The data retrieved from AgroMeteo show a medium temperature during the study period of 22.9 °C, with a minimum of 12.8 °C and a maximum of 32 °C. Relative humidity ranged between 16.8–100%, with a mean of 65.7%. Only 6 days of rain with a total of 80.6 mm (Additional file 1: Figure S4) were recorded during the study period. The weather station in Coldrerio did neither record wind direction nor wind speed.

In Lugano, the temperature from the data loggers ranged between 10.2–55 °C, with a mean of 22 °C. The data obtained with our weather station showed a mean temperature of 20 °C with a minimum of 7.9 °C and a maximum of 30.9 °C. Relative humidity ranged between 19.0–97.0%, with a mean of 73%. Over the whole study period there was less than 30 mm rain (Additional file 1: Figure S4). The prevailing winds were from the South-East.

## Discussion

Our aim was to measure how far emerging *Ae. albopictus* fly to seek a host or a mate, how long they survive and what the mosquito density is in an area of intensive mosquito surveillance and control. These parameters are important for the risk assessment of disease transmission and for making informed choices to minimise that risk. We found that 77.5% of the recaptured *Ae. albopictus* individuals flew further than 250 m, the limit that is usually deemed sufficient for vector containment. The average age of females and males was 8.6 and 7.8 days in Coldrerio and Lugano, respectively, while the estimated mosquito population densities were 134 mosquitoes/ha in Coldrerio and 767 mosquitoes/ha in Lugano.

The results of the present study confirm that active dispersal of host-seeking female and male *Ae. albopictus* may be greater than generally assumed. Previous studies have suggested that the maximum flight range is only around 100 to 200 m [44–46] and concluded *Ae. albopictus* to have weak flight capacity [47]. This conclusion has become the general notion on which many guidelines for the surveillance of invasive mosquitoes are based, for example it is recommended to spray insecticides within a range of 100–300 m around the residence of a viremic person [18, 19]. In contrast, others have also pointed out that *Ae. albopictus* females and males may actually fly beyond 1 km [36, 48], and flight performance experiments under laboratory conditions found that well-nourished females even fly as far as 8.6 km without taking another sugar- or blood meal [15]. These studies, together with the present results, suggest that *Ae. albopictus* is, indeed, a rather “strong” flyer, and that *Ae. albopictus* flight distances may not be limited by its flight ability but are rather dependent on other parameters.

The variations observed in the flight range are likely to be an outcome of a mix of environmental factors such as landscape features, availability of hosts, breeding sites and sugar sources [44, 45, 49], but the outcome of an MRR study also depends on a series of factors related to the experimental procedure. For example, while larval growth conditions have been shown to directly influence mosquito dispersal [22], in most MRR studies in *Ae. albopictus*, the mosquitoes released were sourced from laboratory colonies (Table 1).

In some cases, eggs were field-collected but still reared in the laboratory [50] or in cages placed in the field [45]. Here, we have tried to overcome this drawback by using a set-up that exploits the natural emergence patterns of adults from pupae. Another point of consideration is how the mosquitoes are marked. Ideally the marking method should neither alter the behaviour nor the survival rate of the insects, should be easy to apply, cost-effective and environmentally friendly. Yet, in previous studies, the mosquitoes were frequently removed from a holding cage with a mouth aspirator and then marked individually [51, 52], or in batches, with a pipette releasing a cloud of colour pigment particles [44, 50] or by aspirating them into dusted paper cups [45, 49] or cages [46, 48]. While being time-consuming these methods seem far from ideal in many respects.

In order to minimise any bias due to direct handling of mosquitoes, we decided to deploy self-marking units. In addition, this method is cost-effective when compared to other innovative marking methods such as the use of stable isotopes [36], as the construction of the units requires only few materials and the units are simple to build. Additionally, it is difficult to know the number of mosquitoes released when using a stable isotope and there is still a restrictive number of isotopes available that limit the use of this technology. Another advantage of the self-marking unit is that the mosquitoes are steadily released over a longer period of time (i.e. 16 days in the present study), mimicking a more natural emergence pattern. In contrast, in previous MRR studies, mosquitoes were released in large numbers from a central point at once, a factor which is likely to have influenced their dispersal pattern as dispersal has been shown to be density-dependent [34, 53].

Another caveat of previous studies is that trap densities tended to be higher closer to the release point, increasing the probability of mosquitoes to be recaptured at closer distances and preventing them from flying further away [36]. Trap position and density may also have a strong influence on the results of MRR studies. Ideally, the traps should be randomly distributed with an equal density across the study area. We, too, intended to have an even trap density, but the numbers of households with a power source to run the trap were limited. In some studies [50, 54], the maximum distance flown corresponds to mosquitoes captured at the outer edge of the trapping area. Guerra et al. [55] have also noted that study areas are frequently too small to report maximum flight ranges. To some extent this is also a limitation of the present study as we have still recaptured mosquitoes in the outermost traps. However, we may argue that the relative densities were substantially lower in the outer annulus, as compared to the inner annuli, suggesting that we were able to cover most of the potential flight range. The recapture rate in Coldrerio is in line with previous studies, where the corrected chance to recapture a marked mosquito was 9.3% while the recapture rate was relatively low in Lugano (2.1%). The contradicting results may be explained, among other factors, by differences in the landscape of the two study sites and the number of traps competing with human hosts. Coldrerio is located in a rural area with a lower human population density, has lower buildings and a road system that is more open. Together, these factors might have contributed to an increased recapture rate in Coldrerio as compared to Lugano.

According to the Lincoln formula, the estimated mosquito densities in the two study sites were 134 and 767 mosquitoes/ha in Coldrerio and in Lugano, respectively. The Lincoln estimate may be an overestimation of the true population size [45], especially in Lugano where the chance to capture a mosquito may be considered lower due to the landscape of the city environment. Yet, the figure is still informative as a comparative index. When comparing our results to similar estimates from other MRR studies in Europe [45, 56], where densities were between 50–236 adult mosquitoes/ha, our results suggest rather high population densities.

Female survival is a highly informative parameter for the risk assessment of disease transmission in a given area [57]. The daily survival rates calculated in this study are comparable with results previously reported [28, 44, 46, 50]. As observed in the present study, male survival rates have previously also been reported to be lower than for females [28], although the differences in the present study was rather small. The oldest mosquito recaptured in our study was a female with an age between 19–23 days after release, 805 m away from the marking units. In a laboratory infection study, Heitmann et al. [58] found that European *Ae. albopictus* show high transmission rates for chikungunya at temperatures of 18 °C. For both sites in our study more than 14 consecutive days had an average temperature of 18 °C or above (Additional file 1: Figure S4), suggesting that conditions in Ticino seem favourable for a chikungunya transmission during the summer months, even more so as extrinsic incubation period of chikungunya virus in *Ae. albopictus* is only a few days [41, 59].

## Conclusions

Self-marking units are an effective tool to mark mosquitoes with the main advantage of using wild individuals and a simple marking process that does not impact mosquito survival. We, therefore, recommend the use of the self-marking units for future MRR studies. Using this approach, we found that mosquitoes survive long enough to potentially transmit arboviral disease in our study area. Our results also suggest that host-seeking *Ae. albopictus* females may travel further than previously assumed for European mosquito populations. This finding has direct implications for vector control as emergency treatments around positive cases, as well as surveillance and control around detections of new infestations, might need to be extended beyond the usual recommended range of just a few hundred metres. The extensive survival rates, together with the long distances travelled, underlines the importance of dealing with this highly invasive vector in a coordinated manner across municipalities and country boundaries.

## Supplementary information

**Additional file 1: Figure S1.** Modification of the BG-Sentinel trap. A cylinder-shaped chicken wire (34 × 10 cm) supports the two sticky cards inserted into the BG-Sentinel catch bag to avoid colour cross-contamination between mosquitoes. **Figure S2.** Performance of self-marking units with wild *Ae. albopictus*. Two colours, pink and yellow, were examined for their marking success and their impact on mosquito survival, and compared to a negative control (i.e. cheese cloth without fluorescent dust). **a** Marking success with the two different colours. While the marking success for yellow was very high, the success rate for pink was lower. **b** The survival of *Ae. albopictus* did not differ, neither between colours nor between a colour and the negative control (*χ*^2^ = 2, *df* = 2, *P* = 0.133). The reported male to female ratio is 1:6 and the survival of male and female *Ae. albopictus* did not differ (female *χ*^2^ = 4, *df* = 2, *P* = 0.1, male *χ*^2^ = 1.6, *df* = 2, *P* = 0.4). **Figure S3.** Total number of mosquito collected over the study period. The numbers on top of the bar represents the number of marked mosquitoes. **Figure S4.** Variation of daily mean temperature, mean relative humidity and precipitation during the study period in the two study sites. The blue bars show the daily precipitation in mm per day. **Table S1.** Number of recaptured *Ae. albopictus* during the two MRR studies in the annuli used for MDT calculation.

## Data Availability

The datasets supporting the conclusions of this article are included within the article and Additional file 1.

## Abbreviations

BG: Biogents
FR: flight range
MDT: mean distance travelled
MAX: maximum distance travelled
MRR: mark-release-recapture
