# Supplementary material for: Active dispersal of Aedes albopictus: a mark-release-recapture study using self-marking units
Source: Parasit Vectors. 2019 Dec 12;12:583. doi: 10.1186/s13071-019-3837-5 (PMC6909613; doi:10.1186/s13071-019-3837-5)
Supplement: Supplementary file 1 — Additional file 1: Figure S1. Modification of the BG-Sentinel trap. A cylinder-shaped chicken wire (34 × 10 cm) supports the two sticky cards inserted into the BG-Sentinel catch bag to avoid colour cross-contamination between mosquitoes. Figure S2. Performance of self-marking units with wild Ae. albopictus. Two colours, pink and yellow, were examined for their marking success and their impact on mosquito survival, and compared to a negative control (i.e. cheese cloth without fluorescent dust). a Marking success with the two different colours. While the marking success for yellow was very high, the success rate for pink was lower. b The survival of Ae. albopictus did not differ, neither between colours nor between a colour and the negative control (χ2 = 2, df = 2, P = 0.133). The reported male to female ratio is 1:6 and the survival of male and female Ae. albopictus did not differ (female χ2 = 4, df = 2, P = 0.1, male χ2 = 1.6, df = 2, P = 0.4). Figure S3. Total number of mosquito collected over the study period. The numbers on top of the bar represents the number of marked mosquitoes. Figure S4. Variation of daily mean temperature, mean relative humidity and precipitation during the study period in the two study sites. The blue bars show the daily precipitation in mm per day. Table S1. Number of recaptured Ae. albopictus during the two MRR studies in the annuli used for MDT calculation. [file 13071_2019_3837_MOESM1_ESM.docx]

**Additional file 1**


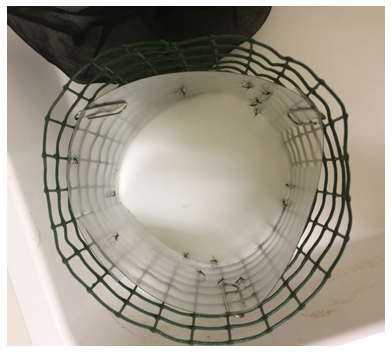


**Additional file 1: Figure S1** **Modification of the BG-Sentinel trap:** a cylinder-shaped chicken wire (34 cm x 10 cm) supports the two sticky cards inserted into the BG-Sentinel catch bag to avoid colour cross-contamination between mosquitoes.

**b**

**a**


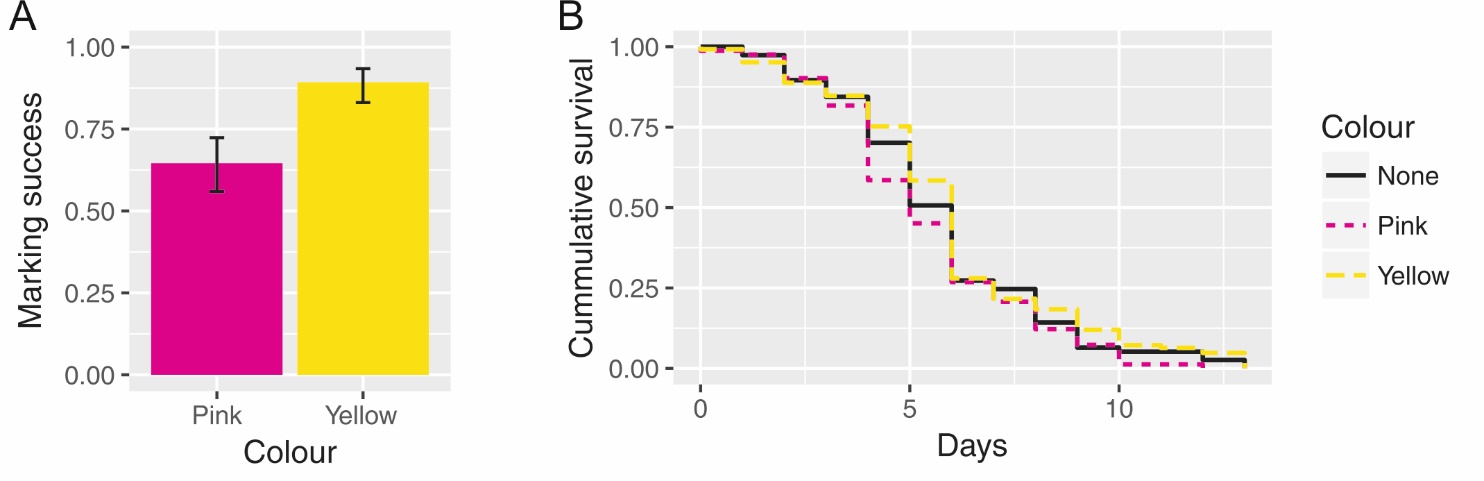


**Additional file 1: Figure S2** **Performance of self-marking units with wild *Ae. albopictus***. Two colours, pink and yellow, were examined for their marking success and their impact on mosquito survival, and compared to a negative control (i.e. cheese cloth without fluorescent dust). **a** Marking success with the two different colours. While the marking success for yellow was very high, the success rate for pink was lower. **b** The survival of *Ae. albopictus* did not differ, neither between colours nor be­tween a colour and the negative control (*χ^2^*=2, *df*=2, *p*=0.133). The reported male to female ratio is 1:6 and the survival of *Ae.albopictus* male and female did not differ (female *χ^2^*=4, *df*=2, *p*=0.1, male *χ^2^*=1.6, *df*=2, *p*=0.4)


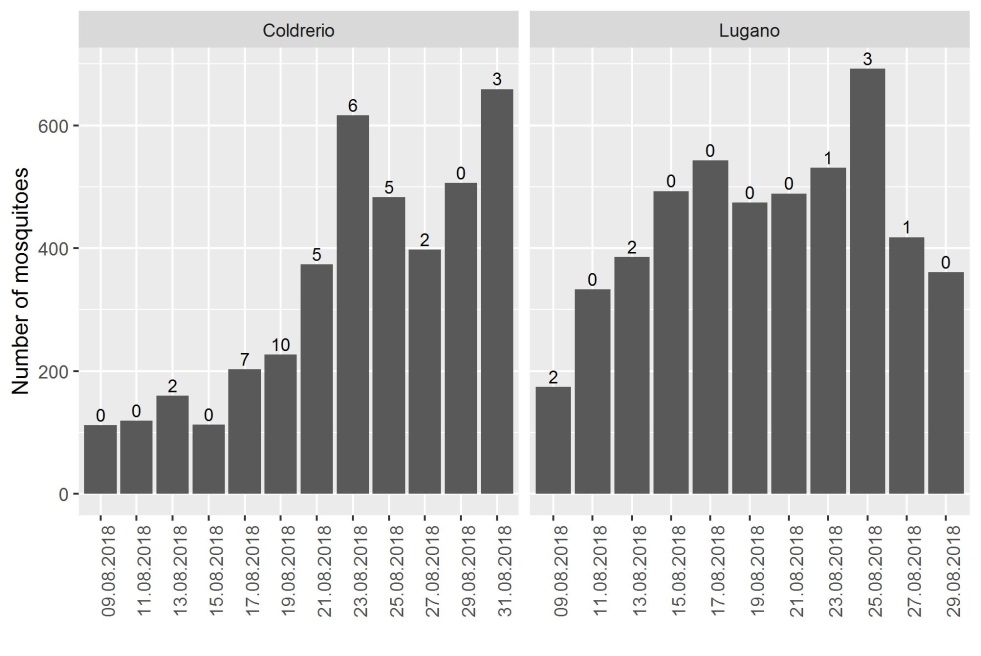
 **Additional file 1: Figure S3** **Total number of mosquito collected over the study period.** The numbers on top of the bar represents the number of marked mosquitoes.


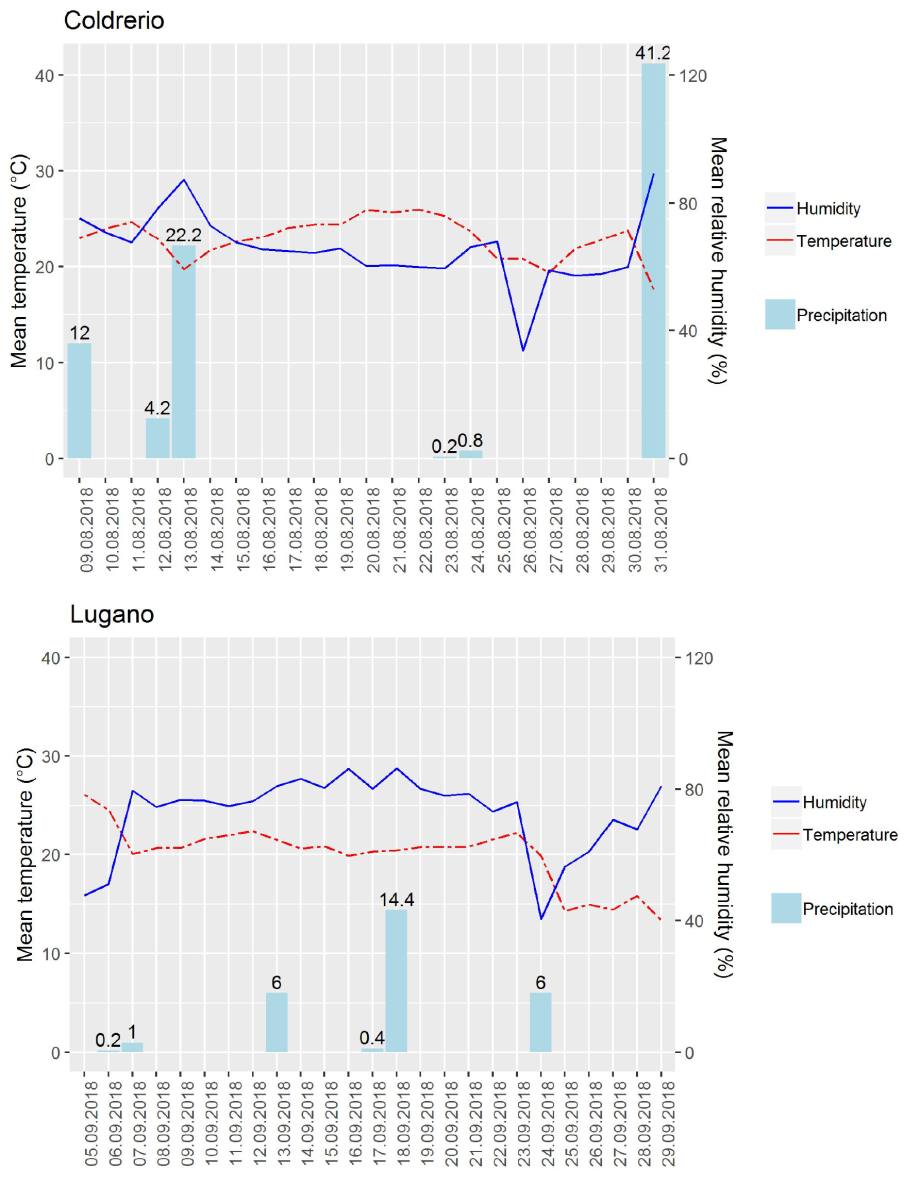


**Additional file 1: Figure S4**  **Variation of daily mean temperature, relative humidity and precipitation during the study period in the two study sites.** The blue bars show the daily precipitation in mm per day.

**Additional file 1: Table S1.** Number of recaptured *Ae. albopictus* during the two MRR studies in the annuli used for MDT calculation.

| **MMR1: Coldrerio** | | | | | | | | | | | | | | | | | | | | | | |
| --- | --- | --- | --- | --- | --- | --- | --- | --- | --- | --- | --- | --- | --- | --- | --- | --- | --- | --- | --- | --- | --- | --- |
| **Meters from releasing (m)** | **N of traps** |  |  |  |  |  |  |  |  |  |  |  |  |  |  |  |  |  |  |  |  |  |
|  |  | **Days after release** | | | | | | | | | | | | | | | | | | | |  |
|  |  | 1-4d | | 3-7d | | 5-9d | | 7-11d | | 9-13d | | 11-15d | | 13-17d | | 15-19d | | 17-21d | | 19-23d | |  |
|  |  | ♀ | ♂ | ♀ | ♂ | ♀ | ♂ | ♀ | ♂ | ♀ | ♂ | ♀ | ♂ | ♀ | ♂ | ♀ | ♂ | ♀ | ♂ | ♀ | ♂ |  |
| 0-250 | 4 | 4 | 2 | 1 | 1 | 1 | 1 | 0 | 0 | 0 | 0 | 0 | 0 | 0 | 0 | 0 | 0 | 0 | 0 | 0 | 0 |  |
| 251-500 | 9 | 3 | 0 | 1 | 0 | 0 | 1 | 2 | 1 | 0 | 0 | 0 | 0 | 0 | 0 | 0 | 0 | 0 | 0 | 0 | 0 |  |
| 501-750 | 9 | 1 | 1 | 2 | 1 | 0 | 0 | 2 | 1 | 1 | 0 | 3 | 0 | 2 | 1 | 0 | 0 | 0 | 0 | 0 | 0 |  |
| 751-1000 | 6 | 1 | 0 | 0 | 0 | 0 | 1 | 0 | 0 | 0 | 0 | 2 | 0 | 0 | 0 | 2 | 0 | 0 | 0 | 1 | 0 |  |
| Subtotal |  | 9 | 3 | 4 | 2 | 1 | 3 | 4 | 2 | 1 | 0 | 5 | 0 | 2 | 1 | 2 | 0 | 0 | 0 | 1 | 0 |  |
| Tot | 28 | 12 | | 6 | | 4 | | 6 | | 1 | | 5 | | 3 | | 2 | | 0 | | 1 | |  |
| Number of mosquito recaptured (marked) | | | | | | | | | | | | | | 40 | | | | | | | |  |
| Number of mosquito released | | | | | | | | | | | | | | 427 | | | | | | | |  |
| Number of mosquito captured (total) | | | | | | | | | | | | | | 3970 | | | | | | | |  |

| **MMR2: Lugano** | | | | | | | | | | | | | | | | | | | | | | |
| --- | --- | --- | --- | --- | --- | --- | --- | --- | --- | --- | --- | --- | --- | --- | --- | --- | --- | --- | --- | --- | --- | --- |
| **Meters from releasing (m)** | **N of traps** |  |  |  |  |  |  |  |  |  |  |  |  |  |  |  |  |  |  |  |  |  |
|  |  | **Days after release** | | | | | | | | | | | | | | | | | | | |  |
|  |  | 1-4d | | 3-7d | | 5-9d | | 7-11d | | 9-13d | | 11-15d | | 13-17d | | 15-19d | | 17-21d | | 19-23d | |  |
|  |  | ♀ | ♂ | ♀ | ♂ | ♀ | ♂ | ♀ | ♂ | ♀ | ♂ | ♀ | ♂ | ♀ | ♂ | ♀ | ♂ | ♀ | ♂ | ♀ | ♂ |  |
| 0-250 | 4 | 1 | 0 | 0 | 0 | 0 | 0 | 0 | 0 | 0 | 0 | 0 | 0 | 0 | 0 | 0 | 0 | 0 | 0 | 0 | 0 |  |
| 251-500 | 6 | 0 | 0 | 0 | 0 | 0 | 0 | 0 | 1 | 0 | 0 | 0 | 0 | 0 | 0 | 0 | 1 | 1 | 0 | 0 | 0 |  |
| 501-750 | 10 | 0 | 0 | 0 | 1 | 0 | 0 | 0 | 1 | 0 | 0 | 0 | 0 | 0 | 0 | 0 | 0 | 0 | 0 | 0 | 0 |  |
| 751-1000 | 6 | 0 | 1 | 1 | 0 | 1 | 0 | 0 | 0 | 0 | 0 | 0 | 0 | 0 | 0 | 0 | 0 | 0 | 0 | 0 | 0 |  |
| Subtotal |  | 1 | 1 | 1 | 1 | 1 | 0 | 0 | 2 | 0 | 0 | 0 | 0 | 0 | 0 | 0 | 1 | 1 | 0 | 0 | 0 |  |
| Tot | 26 | 2 | | 2 | | 1 | | 2 | | 0 | | 0 | | 0 | | 1 | | 1 | | 0 | |  |
| Number of mosquito recaptured (marked) | | | | | | | | | |  | |  | | 9 | | | | | | | |  |
| Number of mosquito released | | | | | | | | | |  | |  | | 425 | | | | | | | |  |
| Number of mosquito captured (total) | | | | | | | | | |  | |  | | 4894 | | | | | | | |  |
